# Frequency, pattern and predictors of cognitive impairments in patients with Parkinson’s disease using the Community Screening Instrument for Dementia

**DOI:** 10.3389/fnhum.2023.1126526

**Published:** 2023-06-27

**Authors:** Ewere Marie Ogbimi, Fatai Momodu Akemokwe, Olubunmi Ogunrin

**Affiliations:** ^1^(formerly Neurology Unit, Department of Medicine, University of Benin Teaching Hospital, Benin City, Nigeria) Neurology Unit, Department of Medicine, Delta State University, Abraka, Nigeria; ^2^(formerly Neurology Unit, Department of Medicine, University of Benin Teaching Hospital, Benin City, Nigeria) Department of Neurology, University of Kentucky, Lexington, KY, United States; ^3^(formerly Neurology Unit, Department of Medicine, University of Benin, Benin City, Nigeria) Neurology Department, Neuroscience Directorate, Royal Stoke University Hospital, Stoke on Trent, United Kingdom

**Keywords:** apraxia, cognition, memory, Nigeria, Parkinson’s disease, CSID

## Abstract

**Background:**

Parkinson’s disease (PD) is a chronic neurodegenerative disorder complicated by cognitive dysfunctions which are associated with increased caregiver burden, pressure on community health facilities, and mortality in affected patients. Most of the data concerning cognitive dysfunctions in PD are from studies conducted in Europe and North America, but there is paucity of data from Sub-Saharan Africa.

**Objective:**

The objective of this study is to determine the frequency, pattern and predictors of cognitive impairments amongst patients with Parkinson’s disease.

**Materials and methods:**

This was a cross sectional case control study carried out at a tertiary health facility in South-south Nigeria. Participants with PD were consecutively recruited from the neurology outpatient clinics. Demographic and disease-specific data were obtained with the use of a pre-tested questionnaire. Cognitive performance of thirty patients with PD were compared with thirty demographically matched controls using the Community Screening Instrument for Dementia (CSID). CSID was already validated among Nigerians.

**Results:**

The frequency of cognitive impairment using the CSID was 50% for PD patients (3.3% for controls). Poor cognitive performance was observed across several cognitive domains including language, executive dysfunction, psychomotor speed, and constructional apraxia among PD patients. The independent predictors of the overall cognitive impairment in patients with PD determined by logistic regression analysis include recall deficiency (*p* = 0.007), impairment with naming (*p* = 0.044), apraxia (*p* = 0.003), Hoen&Yahr staging (*p* = 0.046), UPDRS score (*p* = 0.015) and age at presentation (*p* = 0.014).

**Conclusion:**

Cognitive impairments occur more frequently in patients with PD compared to controls. This study also demonstrated the predictive role of severity of disease based on Hoehn &Yahr staging and UPDRS score, and presence of recall deficiency, poor naming ability and apraxia.

## Introduction

Parkinson’s disease (PD) is a chronic debilitating disease, complicated by cognitive dysfunction and associated with increased caregiver burden, pressure on community health facilities and significant mortality (Okubadejo et al., 2006; Akinyemi et al., 2008). Most of the data concerning cognitive dysfunction in PD are based on studies done in Europe and North America. There is paucity of data on cognitive impairments among PD patients in Sub-Saharan Africa. Knowledge of the pattern of cognitive dysfunction and predictive factors perculiar to different geographical regions may be important in early identification of this complication and ensuring appropriate channeling of resources into caring for patients with PD.

Cognition refers to all mental activities associated with thinking, knowing, remembering and communicating. It embodies solving problems, making decisions, mental grouping of objects and forming judgments (Lezak et al., 1995). The major classes of cognition include reception (ability to select, acquire, classify and integrate information), memory and learning (which involve information storage and retrieval), thinking (involving mental organization and recognizing the information), and expressive functions (which involve speaking, drawing, writing, physical gestures, facial expressions and movements) (Lezak et al., 1995).

The prevalence of cognitive impairments in PD has been reported in several developed countries. A systematic review of 27 studies by Cummings reported an average prevalence of 40% while Aarsland et al. (2007) in their systematic review found the prevalence of cognitive impairments in PD to range between 24 and 31% (Cummings, 1988a; Aarsland and Kurz, 2010). The UK CamPaIGN study obtained a prevalence of 36%, while Muslimovic et al. (2005) found a prevalence of 24% in a cohort of newly diagnosed PD patients in the Netherlands (Foltynie et al., 2004). One of the few studies from the sub-Saharan Africa by Osuntokun and Bademosi (1979) found a frequency of 5% but a more recent study by Akinyemi et al. (2008) reported a frequency of 21.6%.

Several studies, mostly from developed countries, have described cognitive domains affected in PD. Such domains include memory, language functions, attention and concentration, mental speed, motor fluency and praxis. As regards the predictors of cognitive dysfunction in PD patients, most studies agreed on the role of disease severity and presence of dyspraxia. There are conflicting reports on the predictive role of age at onset, duration of disease and level of education of the affected patients on cognitive impairment. It has also been argued that since PD is common among the elderly people (>65 years), the age-related cognitive impairment may be contributing to the cognitive dysfunction in PD thereby confounding the significance of age at onset of the disease. In the light of this, there is need to evaluate the disease and patient variables that can predict development of impaired cognition in PD.

The dearth of information on the pattern and predictive factors of cognitive dysfunction in sub-Saharan African region prompted this study.

Therefore, this study sought to determine the frequency and pattern of cognitive dysfunction amongst patients with PD presenting at a tertiary health facility in south-south Nigeria and compared the performance of the PD patients with demographically matched subjects without PD (controls). In addition, we assessed the contributory roles of patient- and disease- specific variables to the development of cognitive impairments in PD patients.

## Patients and methods

### Study site

The study was carried out in the Neurology outpatient’s clinic of a tertiary health facility located in the cosmopolitan south-south Nigeria. The health facility is situated in the capital of Edo State and serves as the main referral centre for the state and four neighboring states.

### Study population

Patients with PD, without diabetes mellitus or hypertension, presenting for treatment on an out-patient basis at the medical outpatient clinic of the hospital. Patients were examined by one of the authors (MO) and clinical diagnosis was made based on the UK Parkinson’s Disease Society(UKPDS) Brain Bank Clinical Diagnostic Criteria (Hughes et al., 1992; Litvan et al., 2003).

### Inclusion criteria for patients

1. Patients with a clinical diagnosis of PD based on the UKPDS Brain Bank Clinical Diagnostic Criteria (Hughes et al., 1992; Litvan et al., 2003).
2. Patients aged 18 years and above.
3. Patients who gave informed verbal or written consent.
4. Absence of severe functional impairment or severe medical illness that would interfere with the ability to perform the study evaluations.

### Exclusion criteria for patients

1. Patients who do not meet the criteria for clinical diagnosis of PD.
2. No informed verbal or written consent.
3. Patients less than 18 years of age.
4. History of cerebrovascular disease, significant head injury, previous encephalitis, drug or alcohol abuse.
5. Patients on neuroleptic treatment within the preceding 6 months of onset of symptoms.
6. Features of parkinsonism plus syndromes such as early dysautonomia, early cognitive impairment before 1 year of onset, supranuclear gaze palsy, cerebellar signs, or negative response to levodopa (L-dopa).
7. PD patients with co-morbidities capable of causing cognitive impairment (e.g., chronic kidney disease, cardiac disease, liver disease, and diabetes mellitus).
8. Hoehn and Yahr (Osuntokun et al., 1987) stage five patients with PD who were largely home-bound.
9. Inability to complete the study.

### Inclusion/exclusion criteria for controls

Normal healthy individuals who fulfilled the above inclusion criteria except that they did not have features of PD or Parkinson’s Syndrome, were recruited from the hospital staff and general population and freely volunteered to be part of the study. They were age, sex and level of education matched with PD patients.

### Sampling technique

Consecutive patients with features of PD who met the inclusion criteria were recruited at presentation in the neurology outpatient clinics.

### Sample size

A total of 30 patients were recruited for the study. The minimum sample size of patients required for the study was 28, calculated based on the Kish method (Kish, 1965).


n=z⁢pq2d2=1.96×20.0006×0.9994(0.009)2=28.44

*n* = the desired sample size (when population is greater than 10,000).
z = the standard normal deviation, usually set at 1.96, which corresponds to the 95% confidence level.
*p* = proportion of patients with PD estimated at 59 per 100,000 (De Rijk et al., 1995, 1997; Benito-Leon et al., 2003).
q = 1-p.
d% = absolute deviation from p% that will be tolerated.
(i.e., p% give or take d%).

### Study design

Recruited PD patients with age-, sex, and level of education-matched controls received clinical assessment using standardized questionnaires to document demographic information and disease related variables including primary language used and reading abilities, level of education, medical history and psychiatry history. A detailed neurological examination was performed on all patients to verify the clinical diagnosis of PD. The Hoehn and Yahr (1967) staging scale and motor subscale from the Unified Parkinson’s Disease Rating Scale(UPDRS) (Fahn et al., 1987) were used to determine the stage and severity of the disease. The duration of the disease was defined by the period between the onset of the first symptom as reported by the patient and the time of clinical assessment.

Cognitive performances of recruited study participants (PD patients and controls) were assessed with the Community Screening Instrument for Dementia (CSI-D) (Hall et al., 1993, 1999; Imarhiagbe et al., 2005).

The CSI-D was selected as the cognitive assessment tool because it was previously validated among Nigerians (Hall et al., 1999) and has been used in several other studies (Salawu et al., 2008; Obiabo et al., 2012; Akinyemi et al., 2014; Farombi et al., 2016). This validated version was used for this study.

The CSI-D has two parts; a patient cognitive screen (COGSCORE) in which the subject is asked a series of questions and asked to complete cognitive tasks and a second section completed by a close relative or friend acting as an informant, which includes questions related to functional performance, such as activities of daily living (RELSCORE). The scores for the two sections can be completed to give an overall score that can be used to rank individuals into “probable dementia,” “possible dementia,” and “no dementia” categories (Hall et al., 1999; Obiabo et al., 2012).

All questionnaires used for the study were in English language as most people in the locality speak English.

### Ethical consideration

Ethical approval was obtained from the institutional Ethics and Research Committee before proceeding with the study. Informed written consent was obtained from patients and controls or a reliable relation before recruitment after proper explanation of the study procedure. Recruited study participants were compensated with transport allowance.

### Statistical analysis

Data were analyzed using the Statistical Package for Social Sciences version 16.0 (SPSS) (SPSS, 2007). Demographic characteristics of cases and controls were compared using the Student’s *t*-test for continuous variables, while categorical parameters were analyzed using Chi-squared test. Clinical characteristics of cases were summarized using the mean and standard deviation (SD). Cognitive performances of cases and controls were compared using the Student’s *t*-test and cognitive impairment on the CSID for the purposes of this study was taken as a score below two SD of the mean score of the control group (Salawu et al., 2008), whereas independent predictors of cognitive dysfunction were identified using odds ratios (ORs) from logistic regression models. The confidence interval was set at 95% and a *p*-value of 0.05 or less was considered statistically significant.

## Results

### Demographic data of patients and controls

**FIGURE 1:**
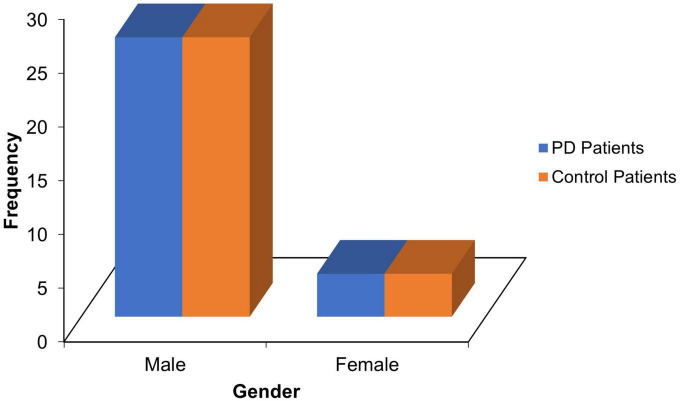
Sex distribution of PD patients and control.

Thirty PD patients and age-, sex- and level of education-matched 30 controls with mean ages of 69.10 ± 7.47 and 68.73 ± 7.06 years, respectively (refer Table 1), and male preponderance(M/F = 6.5:1; see Figure 1) participated in the study.

**TABLE 1:** Demographic data of Parkinson’s disease (PD) patients and control.

Variable	PD patients		Control patients		Test	*P*-value
	**Frequency (%)**	**Mean ± SD**	**Frequency (%)**	**Mean ± SD**		
Age (yrs)	–	69.10 ± 7.47	–	68.73 ± 7.06	t = 0.195	0.846
**Age (yrs)**						
≤65	12 (40.0)	–	10 (33.30)	–	χ^2^ = 0.789*	0.287
>65	18 (60.0)	–	20 (66.7)	–	–	–
**Gender**						
Males	26 (86.7)	–	26 (86.7)	–	χ^2^ = 0.000*	1.000
Females	4 (13.3)	–	4 (13.3)	–	–	–
**Education**						
None	3(10.0)	–	3 (10.0)	–	χ^2^ = 0.000	1.000
Primary	6 (20.0)	–	6 (20.0)	–	–	–
Secondary	10 (33.3)	–	10 (33.3)	–	–	–
Tertiary	11 (36.7)	–	11 (36.7)	–	–	–

*Fisher’s Exact test.

**FIGURE 2:**
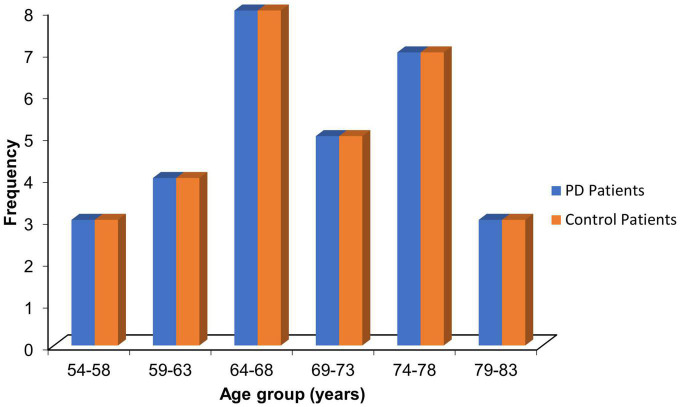
Age distribution of PD patients and control.

The mean age for the PD patients was 69.10 ± 7.47 years, while that of the control was 68.73 ± 7.06 years (*p* = 0.846). Most PD patients (26.7%) were within the 64–68 age group. The proportion of patients and corresponding controls in the stratified age groups is shown in Figure 2.

### Clinical characteristics of the patients with PD

**FIGURE 3:**
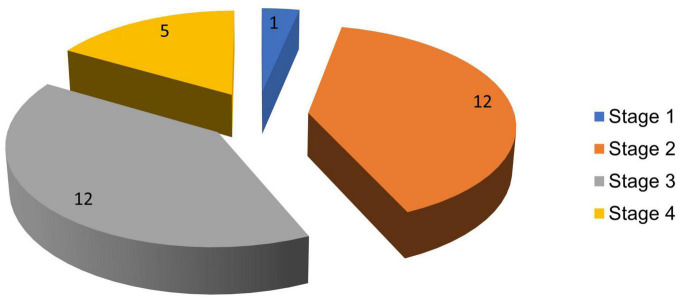
Hoehn and Yahr staging of PD patients.

The highest proportion of PD cases was seen in stages 2 and 3 of the H and Y scale, each comprising 12 (40%) subjects. The lowest proportion was in stage 1 with 1 (3.3%) patient, while stage 4 had 5 (16.7%) patients. Figure 3 reflects the stages of PD in the population studied.

The overall UPDRS score range for the PD patients was 7–64, with a mean of 35.23 ± 14.69. The scores for the males ranged between 7 and 64 with a mean of 35.15 ± 14.25, while those of the females were higher ranging between 22 and 64 with a mean of 35.75 ± 19.81. Table 2 shows the UPDRS rating of the PD patients.

**TABLE 2:** Unified Parkinson’s Disease Rating Scale (UPDRS) rating of Parkinson’s disease (PD) patients.

	*N*	Range	Mean ± SD
UPDRS score	30	7–64	35.23 ± 14.69
Male	26	7–64	35.15 ± 14.25
Female	4	22–64	35.75 ± 19.81

### Cognitive performance among patients with PD and control subjects

The total mean scores on CSID were 57.17 ± 9.19 and 64.7 ± 1.95 for the PD patients and controls, respectively (*p* < 0.001). Fifteen (50%) of the PD patients were cognitively impaired, while one (3.3%) of the control was impaired. Cognitive impairment on the CSID for the purposes of this study was taken as a score below two SD of the mean score of the control group, this corresponded to a score of 38.79. Table 3 shows the performance of the PD patients and the controls.

**TABLE 3:** Performance of patients and controls on Community Screening Instrument for Dementia (CSID).

Variable	PD patients		Control patients		Test	*P*-value
	**Frequency (%)**	**Mean ± SD**	**Frequency (%)**	**Mean ± SD**		
CSID score		57.17 ± 9.19		64.70 ± 1.95	t = 3.924	<0.0001
**CSID**						
Normal	15 (50.0)		29 (96.7)		χ^2^ = 16.705	<0.0001
Impaired	15 (50.0)		1 (3.3)			

The patients with PD performed poorly in most of the cognitive domains compared with control. The exceptions were attention and calculation (*p* = 0.211), orientation in place (*p* = 0.107) and time (*p* = 0.083), three-item recall (*p* = 0.488), definition (*p* = 0.184), and repetition (*p* = 0.161) with no statistical significance. The performance according to domain is reflected on Table 4.

**TABLE 4:** Cognitive performance by domains among patients and controls on Community Screening Instrument for Dementia (CSID).

Domain	Cases *N* = 30 mean (SD)	Controls *N* = 30 mean (SD)	t	df	*P*-value
**CSI-D**					
Attention and calculation	3.83 (0.53)	3.97(0.18)	−1.278	29	0.211
**Orientation**					
Place	5.60 (0.97)	5.90 (0.31)	−1.663	29	0.107
Time	5.60 (0.77)	5.80 (0.51)	−1.795	29	0.083
**Memory**					
3-item recall	2.37 (1.03)	2.57 (0.94)	−0.769	29	0.488
Name recall	1.53 (0.63)	1.87 (0.35)	−2.763	29	0.010*
Short story test	9.80 (2.82)	11.97(0.18)	−4.347	29	0.001*
Past national events	2.57 (0.86)	2.93 (0.25)	−2.164	29	0.039*
Total memory score	18.7 (4.06)	21.53 (0.97)	−3.854	29	0.001*
**Language**					
Naming (visual confrontation)	6.87 (0.35)	7.00 (0.00)	−2.112	29	0.043*
Definition	3.87 (0.35)	3.97 (0.18)	−1.361	29	0.184
Repetition	1.93(0.25)	2.00 (0.00)	−1.439	29	0.161
Comprehension- motor response	4.80 (0.55)	5.00 (0.00)	−1.989	29	0.056*
Language total	19.27 (1.31)	20.33 (0.48)	−4.455	29	0.001*
**Executive function**					
Animal naming	11.27 (3.65)	14.08 (3.36)	−3.566	29	0.001*
Fluency test (1 min)	–	–	–	–	–

*Statistically significant.

### Predictors of cognitive dysfunction

There is a linear relationship between the cognitive performance on the CSID and the H-Y stages. PD patients with more severe disease as reflected in higher H-Y stages performed worse than those with mild disease (*p* = 0.046). Apraxia score was a stronger predictor of the overall performance on CSID (*p* = 0.003).However, the gender, age group, level of education, UPDRS score and disease duration did not significantly predict the performance of PD patients on CSID (*p* > 0.05). Table 5 shows the relationship of the performance of the PD patients on CSID with H &Y staging, gender, age group, level of education, UPDRS, apraxia, and disease duration.

**TABLE 5:** Relationship between Community Screening Instrument for Dementia (CSID) performance H and Y, gender, age group, level of education, Unified Parkinson’s Disease Rating Scale (UPDRS), apraxia, disease duration.

	CSID status		χ^2^	*P*-value	Relative risk	95% CI
	**Normal [F (%)]**	**Impaired [F (%)]**				
**H and Y**						
Stage 1 and 2	9 (60.0)	4 (26.7)	3.394^+^	0.139^+^	4.125	0.883–19.273
Stage 3 and 4	6 (40.0)	11 (73.3)	–	–	–	–
**Gender**						
Female	1 (6.7)	3 (20.0)	0.288*	0.591*	0.464	0.082–2.630
Male	14 (93.3)	12 (80.0)	–	–	–	–
**Age group**						
≤65	5 (33.3)	5 (33.3)	0.000^+^	1.000^+^	1.000	0.219–4.564
>65	10 (66.7)	10 (66.7)	–	–	–	–
**Level of education**						
None	0 (0.0)	3 (20.0)	5.277^+^	0.138^+^	–	–
Primary	3 (20.0)	3 (20.0)	–	–	–	–
Secondary	4 (26.7)	6 (40.0)	–	–	–	–
Tertiary	8 (53.3)	3 (20.0)	–	–	–	–
	**Mean ± SD**	**Mean ± SD**				
UPDRS	30.20 ± 14.08	40.27 ± 13.94	−1.968^‡^	0.059	–	−20.547 to −0.414
Apraxia	1.67 ± 0.49	0.93 ± 0.70	3.317^‡^	0.003	–	0.280–1.186
Disease duration(yrs)	4.29 ± 2.90	3.33 ± 1.91	1.072^‡^	0.293	–	−0.87 to 2.798

*χ^2^ with Yates correction.

^+^Fisher’s Exact test.

^‡^ Student’s *t*-test.

F, frequency.

^†^Row-variable dependent.

CI, confidence interval.

**FIGURE 4:**
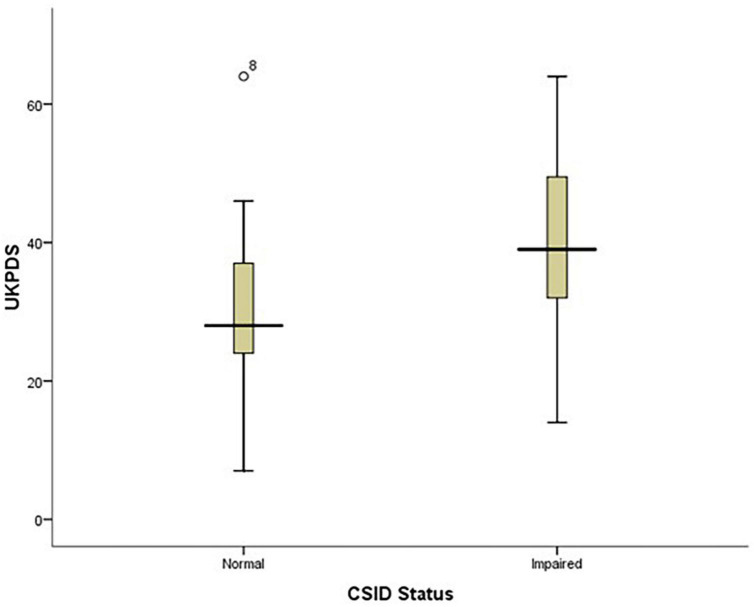
Box plot showing the relationship between patients’ performance on CSID and their UPDRS score.

The findings of this study showed that PD patients with impaired CSID status had higher UPDRS scores than those with normal CSID status (*p* = 0.003) see Figure 4.

**FIGURE 5:**
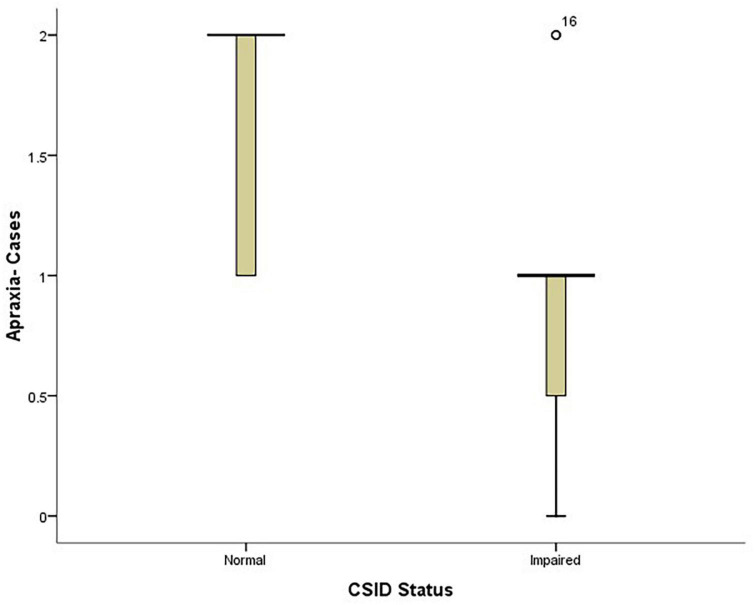
Box plot showing the relationship between patients’ performance on CSID and their apraxia performance.

Also, the PD patients with lower apraxia scores performed worse on CSID (*p* = 0.009) see Figure 5.

The PD patients were shown to have lower scores with increasing age of onset. However, this was not statistically significant with a *P*-value of 0.169.

Recall and naming on the CSID were significant predictors of cognitive dysfunction with significant *P*-value of <0.05. However, education and gait were not statistically significant at *p*-value > 0.05.

## Discussion

This case control study compared the cognitive performances of 30 PD patients with age, sex, and level of education matched controls using the CSID and demonstrated that cognitive impairments are more common in PD, also showed the predictive significance of apraxia, severity of disease based on Hoehn and Yahr staging and UPDRS score, and presence of recall deficiency, and poor naming ability.

The patients were in the sixth decade of life. This may be due to the fact that PD is rare before the age of 50 years with a sharp increase in incidence after the age of 60 years (Ericksen et al., 2005; De Lau and Breteler, 2006). Majority of the patients that presented during this period were males and this is consistent with earlier studies that have reported a higher prevalence of PD in men than in women (Li et al., 1985; Mayeux et al., 1995; Fall et al., 1996; Claveria et al., 2002; Benito-Leon et al., 2003; Okubadejo et al., 2010). It was also observed that most of the PD patients presented in stages 2 and 3 of the Hoehn and Yahr staging, this may be due to the fact that most patients with PD do not present early; probably due to lack of impairment in activities of daily living in the early stage of the disease. The fewer number in the later stages may be because severe cases might have died either from complications of the disease or other age-related illnesses.

The PD cohort studied had significant cognitive impairment as demonstrated in their performances on all the neuropsychological tests used. This is similar to findings in previous studies on cognitive impairment in PD. The frequency of cognitive dysfunction in the PD patients was 50% using the CSID. This is higher than those obtained in other studies (Foltynie et al., 2004; Akinyemi et al., 2008; Aarsland and Kurz, 2010). The variation in prevalence of PD from this study may be attributed to differences in methods of cognitive assessment, operative definition and the study population (Emre, 2003a). This study was however, on cognitive impairment and not just dementia alone.

The CSID assessed cognitive domains of attention and calculation, orientation to time and place, memory, language, executive function, psychomotor speed and constructional apraxia. The overall performance of patients with PD on the test instrument was significantly worse when compared with the performance othe healthy subjects. The CSID demonstrated cognitive dysfunction in the domains of memory, language, executive function and visuospatial dexterity. This is in keeping with earlier studies done (Hall et al., 1993, 1999; Imarhiagbe et al., 2005). The patients overall performance in the memory cognitive domain was poor compared with the controls. Memory deficit in PD is more a problem of retrieval of coded information than a deficit of registration, encoding and storage of information (Tomer and Rey, 1992; Okubadejo et al., 2006; Salmon and Filoteo, 2007). This was demonstrated in this study by the performance of the PD subjects in the name recall, short story test, past national events and overall memory score. Their performance was worse than that of the controls.

Language deficits have been well-described in PD and these include naming difficulties, decreased information content of spontaneous speech, impaired comprehension of complex sentences, as well as verbal fluency (Bayles et al., 1993; Péran et al., 2003). These were exhibited in our cohort of subjects. Confrontational naming defects exhibited by our subjects may be as a result of direct neuronal degeneration associated with hippocampal and limbic deposition of Lewy body pathology in PD (Braak et al., 2003). However, in many described language deficits, the anatomical regions of the brain responsible for language function are not primarily affected by the disease process, rather they may be related to the dysequilibrium syndrome that characterizes PD (Starkstein et al., 1989; Emre, 2003b). In addition, patients with PD may develop a limbic or hippocampal type of amnesia similar to that seen in Alzheimer’s disease. Such subjects are those in whom AD and PD co-exist or those with AD type pathology (Cummings, 1988b).

The patients with PD performed worse than the controls in the tests assessing executive dysfunction such as the animal naming fluency test (1 min) and comprehension-motor response. The timed test offers some quantitative measure of the speed of cognitive processing (reduced in PD). PD patients are impaired in tasks requiring the spontaneous elaboration, maintenance and change of cognitive strategies (Bowen et al., 1975; Matison et al., 1982; Weingartner et al., 1984; Flowers and Robertson, 1985; Taylor et al., 1986; Gotham et al., 1988). The short story test as a test of working memory (a recognized subcategory of executive function) (Baddeley, 1992; D’Esposito et al., 1995) was impaired in the PD subjects.

According to Farina et al. (1994) in PD the dysfunction of the “Complex Loop,” linking pre-frontal lobe to the basal ganglia, is combined with damage to the “Motor Loop,” a functional anatomic circuit connecting the putamen to the supplementary motor area, resulting in disruption in functions of the ascending dopaminergic mesocorticolimbic pathway and frontal cortex responsible for the motor disability and cognitive symptoms. The frontal-like neuropsychological abnormalities observed in PD in another hypothesis was attributed to the loss of dopaminergic neurons in the ventral tegmental area; affecting meso-cortico-limbic pathways, causing dopamine depletion in various cortical areas, including the pre-frontal cortex (Lees and Smith, 1983; Torak and Morris, 1986; Sharpe, 1990).

In this study, certain variables were demonstrated to predict cognitive dysfunction. Although the age groups did not significantly correlate with the CSID, and thus was not related to the level of overall cognitive function. The Campaign cohort study revealed that age greater than 72 years was an independent predictor of cognitive decline in PD patients (Williams-Gray et al., 2009a).

Age at onset of the disease and disease duration were not found to be predictors of cognitive dysfunction. This is in keeping with Hughes et al. (2000) who found no relationship between age at onset of the disease and cognitive decline. Aarsland et al. (2007) however, showed that it is the general effect of age rather than the age of PD onset that contributes most to the incident dementia in patients with PD, while Lyros et al. (2008) showed that the cognitive decline in PD patients might be owing to simultaneous effect of age-related and disease associated neuropathology. This was at variance with Akinyemi et al. (2008) who demonstrated that older age at onset of PD showed significant association with cognitive dysfunction.

Previous studies have shown that the most important clinical predictors of global cognitive decline following correction for age were neuropsychological tasks with a more posterior cortical basis including semantic fluency less than 20 words in 90 s and inability to copy an intersecting pentagon figure, as well as a postural instability gait abnormality (non-tremor dominant) motor phenotype at the baseline assessment (Katzen et al., 1998; Levy et al., 2002; Azuma et al., 2003; Williams-Gray et al., 2009a,b; Aarsland and Kurz, 2010; Taylor et al., 2008). This study also demonstrated that semantic fluency and inability to copy an intersecting pentagon were significant predictors of cognitive dysfunction, in addition recall was also significant, however, was at variance with the previous studies in that gait abnormality was not found to be a significant predictor of cognitive dysfunction.

We demonstrated that greater severity of PD as indicated by higher Hoehn and Yahr stages or higher scores on the UPDRS motor subscales was associated with worse cognitive performance which is in tandem with previous studies (Blessed et al., 1968; Muslimovic et al., 2005; Akinyemi et al., 2008).

There was no significant relationship between years of formal education and cognitive decline. This is in keeping with studies by Akinyemi et al. (2008) and Pai and Chan (2001). However, Glatt et al. (1996) and Green et al. (2002) found an association between lower educational attainment and cognitive decline in subjects with PD.

It is important to mention that there are a few studies that used Mini-Mental State Examination (MMSE) to assess cognitive impairment among Nigerian patients with PD (Ojo et al., 2012; Ojagbemi, 2013), but the CSI-D is more robust in testing for the various cognitive domains and allow for a wider comparison of findings considering its wider acceptance among researchers.

## Limitations

The limitations of this study included potential recall bias and inability to calculate incidence, which are common to case-control studies. However, considering that PD is relatively uncommon in our environment, case control study design was deemed suitable for this research. The sample size is an obvious limitation of our study. Although we calculated the sample size statistically, a larger sample size will provide more generalizable conclusions.

## Conclusion

This study showed that there is a high frequency of cognitive impairment amongst patients with PD compared to demographically matched controls and this worsens with the stage of the disease on Hoehn and Yahr staging and with increasing UPDRS score. Other predictors of cognitive impairment in the PD patients included semantic fluency, apraxia and recall. The pattern of cognitive impairments seen in these patients included memory, psychomotor speed, complex mental speed and visuoperceptual and constructional dexterities.

## Recommendations

All PD patients should be screened for cognitive impairment using neuropsychological tests by their health care providers.

Most of these PD patients are dependent on others for means of livelihood due to incapacitation by the disease. There is need for support groups such as the Parkinson’s society in our local environments.

Further studies are recommended to increase knowledge of this disease and the impact on the cognitive function of affected individuals. This will help in the management of these individuals.

## Data availability statement

The original contributions presented in this study are included in the article/supplementary material, further inquiries can be directed to the corresponding author.

## Ethics statement

The studies involving human participants were reviewed and approved by University of Benin Teaching Hospital Research Ethics Committee, Benin City, Nigeria. The patients/participants provided their written informed consent to participate in this study.

## Author contributions

EO and OO conceptualized, designed, and approved the manuscript. FA analyzed the data and approved the manuscript.
